# Anomalous Subarcuate Canal and Artery: A Rare Anatomical Variation

**DOI:** 10.22038/IJORL.2022.61681.3120

**Published:** 2022-05

**Authors:** Mahendra Chouhan, Payal Kumbhat

**Affiliations:** 1 *Department of Otorhinolaryngology and Head and Neck Surgery, Dr. S.N. Medical College, Jodhpur, Rajasthan, India.*

**Keywords:** Anomalous, Subarcuate canal, Subarcuate artery

## Abstract

**Introduction:**

An aberrant or anomalous subarcuate artery and its canal is an extremely rare and clinically significant finding. If accidentally nicked or injured, it can cause inadvertent hemorrhage and obscure the surgical field.

**Case Report:**

We present a case of 21 year old made with Chronic Otitis Media of the right ear who was incidentally diagnosed with a dilated subarcuate canal and an aberrant subarcuate artery atypically associated with lateral semicircular canal and facial nerve.

**Conclusions:**

Prior knowledge of this symptomatically dormant variation is important, particularly in retrofacial tympanomastoidectomy and cerebello-pontine angle tumor surgeries. Pre-operative temporal bone scans are advisable in such cases.

## Introduction

The radiological anatomy of temporal bone has been extensively studied, but very little has been reported about the petromastoid canal, also known as the subarcuate canal. It is wider and voluminous in infants and young children. In adults, it regresses in size becoming either invisible or a barely perceptible radiolucent line (1). Therefore, it is often either overlooked or confused for a fracture line during routine interpretation of the temporal bone scans. 

The subarcuate canal is a bony canal connecting the posterior cranial fossa with mastoid air cells harboring the subarcuate artery and vein. It arises from the subarcuate fossa, localized on the posterior surface of the petrous bone, runs superiorly and laterally to pass between the crus of superior semicircular canal, and terminates in the mastoid air cells. It supplies the bony labyrinth, facial canal, and mastoid air cells. It can be a potential pathway for the spread of infection to posterior cranial fossa. Any variation from its normal course makes it prone to injury and resultant intra-operative hemorrhage.

We present a case of anomalous course of subarcuate artery along with enlarged subarcuate canal and its atypical association with lateral semicircular canal and facial nerve. After a thorough review, we conclude that this is highly infrequently reported in otolaryngology literature and of significant value in retro-facial mastoid exploration and cerebello-pontine angle tumor surgeries (2,3).

## Case Report

**Fig 1:**
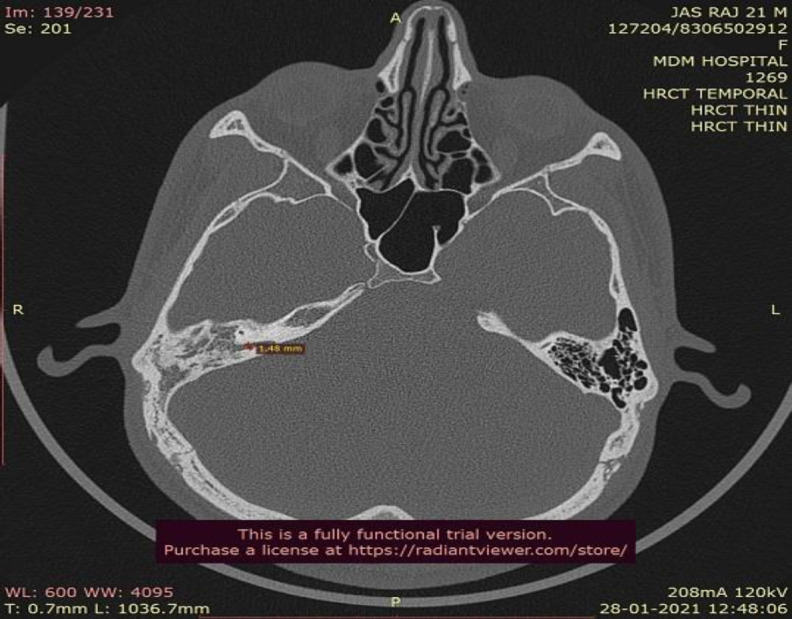
HRCT Temporal Bone scan showing enlarged petromastoid (subarcuate) canal

**Fig 2:**
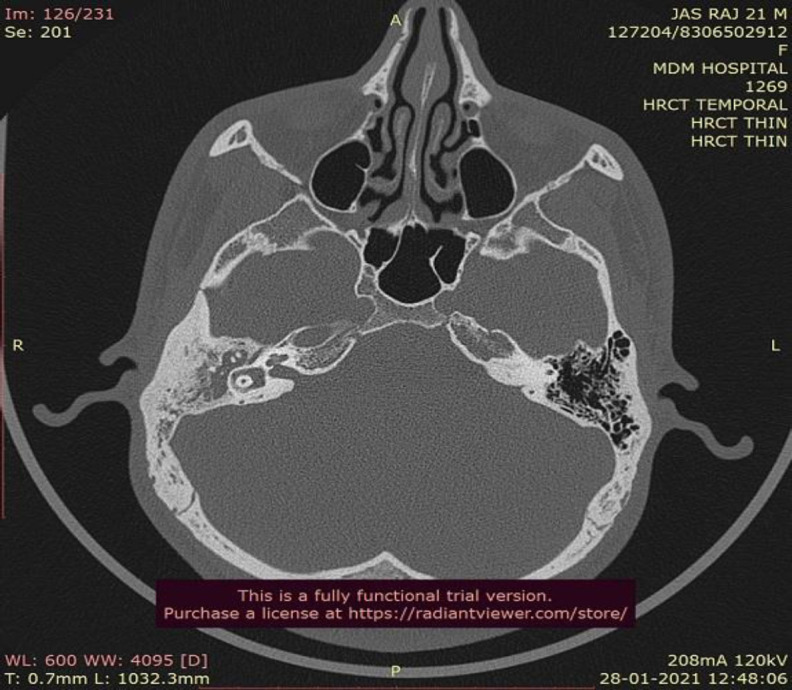
aberrant subarcuate artery passing through the arches of lateral semicircular canal

**Fig 3:**
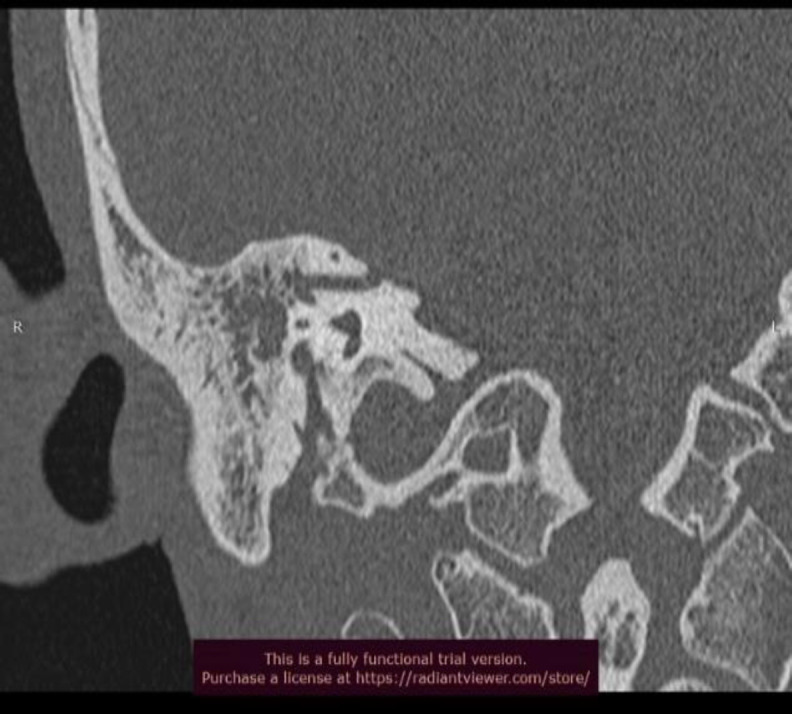
Coronal section of HRCT Temporal Bone showing dilated and anomalous petromastoid (subarcuate) canal

A 21 year old male patient presented to us in the outpatient department with complaints of discharging right ear for 2 years and associated hearing loss since the last 1 year. He also gave a history of intermittent non-pulsatile tinnitus in the right ear which was spontaneously resolved within the last 1 year. On oto-endoscopic examination, his right tympanic membrane showed Sade's grade 2 pars tensa retraction in the posterosuperior quadrant with a non self-cleaning retraction pocket. His tunning fork test revealed a severe conductive hearing loss in the right ear which was confirmed by pure tone audiometry. He had no relevant medical or surgical history and all his routine hematological tests were within normal limits. A high-resolution computed tomography scan of temporal bone was obtained for further evaluation. It was suggestive of soft tissue density in the mastoid air cell system and aditus along with ossicular chain erosion. It also revealed an enlarged subarcuate canal with a width consistently greater than 1 mm, approximately 1.48mm in its greatest transverse dimension. It also showed aberrant subarcuate artery arising from subarcuate fossa, running posterolaterally to pass between the crus of superior semicircular canal. It then turned caudally with a bend and ran through the arches of lateral semicircular canal. From here, it ran adjacent to the mastoid segment of facial nerve separated only by a thin bony ledge. This patient was diagnosed with Chronic Otitis Media with active mucosal disease and operated on for Intact Canal Wall Mastoidectomy.(Fig1,2,3)

## Discussion

The petromastoid canal, when first described by Mouret and Rouviere in 1904, was thought to be an inconstant structure (4). With the development of imaging techniques and the introduction of high resolution computed tomography scans, it began to be seen in all temporal bones (5). It is very voluminous in fetal life. In the first 5 years of life, it begins to regress in size and attains a canaliculus state (6). Until adulthood, when it achieves a roughly constant diameter ranging from 0.5mm to 1mm (7). The canal, also known as the subarcuate canal, is lined by the dura and harbors subarcuate artery and vein.

Mazzoni described the subarcuate artery to arise from an arterial loop lying inside the internal auditory canal or at its porus or in the cerebello-pontine space. It is a branch of anterior-inferior cerebellar artery (80 percent), accessory anterior-inferior cerebellar artery (17 percent), or rarely posterior-inferior cerebellar artery (3 percent). Customarily, the artery enters the petrous bone at the subarcuate fossa. After entering it runs straight in a lateral, posterior, and slightly superior direction. It passes through the bone under the superior semicircular canal and close to the non-ampullary end of the posterior semicircular canal and then divides into two terminal branches medial to the antral wall and above the lateral semicircular canal (8,9).

In its intra-petrous course, the subarcuate artery is divided into three segments: proximal, intermediate, and distal; supplying the otic capsule, labyrinth, vestibule, fallopian canal, and the mastoid air cell system. These segments also form vascular anastomoses with the internal auditory artery, superficial petrosal artery, and the stylomastoid artery respectively (8). The petromastoid canal, along with its contents, is fairly constant in its origin. But, its course may vary owing to the embryological development of vessels and their various anastomoses. However, literature reporting any such anatomical variation is extremely sparse. Our study is only the third of its kind in world literature. Such variable course puts the vessel at the risk of injury thereby resulting in hemorrhage. This can be managed by coagulation of the vessel. 

## Conclusion

The subarcuate artery is not an essential vessel and its coagulation would not be of any major consequence. But, it is imperative that an otologic surgeon is aware of its anomalous course to avoid any untoward hemorrhage and resultant obscurement of the surgical field.
